# Machine Learning Model to Predict Iodine Contrast Media-related Acute Adverse Reaction in Patients without a Similar History for Enhanced CT

**DOI:** 10.2174/0115734056436322251022040623

**Published:** 2025-10-27

**Authors:** Ke-xin Jiang, Wen-yan Liu, Yang Xu, Kun-hua Li, Fang Wen, Rong Zhou, Shi-lan Xiang, Da-jing Guo, Tian-wu Chen, Xiao-lin Wang

**Affiliations:** 1 Department of Radiology, The Second Affiliated Hospital of Chongqing Medical University, No.74 Linjiang Road, Yuzhong District, Chongqing 400010, China; 2 Department of Radiology, Chongqing University Three Gorges Hospital, 165# Xincheng Street, Wanzhou District, Chongqing 404100, China; 3 Department of Radiology, Yongchuan Hospital of Chongqing Medical University, 439# Xuanhua Street, Yongchuan District, Chongqing 402160, China; 4 Department of Radiology, Chongqing University Qianjiang Hospital, 360# Zhengzhou South Street, Qianjiang District, Chongqing 409000, China

**Keywords:** Computed tomography, Iodine contrast media, Acute adverse reaction, Machine learning, Risk prediction, Adverse drug reactions

## Abstract

**Introduction:**

The objective is to develop and compare risk prediction models for Iodine Contrast Media (ICM)-related Acute Adverse Reactions (AAR) in patients without a prior history of such reactions, and to construct a nomogram based on the superior model.

**Methods:**

546 patients without a history of ICM-related AAR who underwent ICM administration during CT contrast-enhanced scan were retrospectively enrolled, and divided into training (n=234), test (n=101), and external validation (n=211) sets. Clinical, medication information, and environmental factors were collected. Features were selected by univariate logistical analysis and least absolute shrinkage and selection operator, and four Machine Learning (ML) models, including Logistic Regression (LR), decision tree, k-nearst neighbors and linear support vector classification were used to construct ICM-related AAR risk prediction models were developed and evaluated using AUC, accuracy and F1 score. A nomogram was constructed based on the superior model.

**Results:**

History of ICM exposure and allergy due to other factors, hypertension, type of ICMs, ICM dose, oral metformin, hyperglycaemia, and glomerular filtration rate were selected for modeling (all *p* < 0.05). The LR model demonstrated superior performance, with AUCs of 0.894 (test set) and 0.814 (external validation), and was used to construct a clinically applicable nomogram.

**Discussion:**

The LR-based model effectively predicts ICM-related AAR risk using readily available clinical variables. It offers a practical tool for identifying high-risk patients prior to ICM administration, facilitating preventive measures.

**Conclusion:**

LR can predict the risk of ICM-related AAR well in patients without a history of ICM-related AAR, and the corresponding nomogram is provided.

## 1 INTRODUCTION

Iodine Contrast Media (ICM) is a substance used in CT contrast-enhanced examination to improve the contrast of tissues on images, making the display of lesions clearer [1]. With the continuous popularization in clinical applications, the incidence of adverse drug reactions has also increased [2, 3], especially Acute Adverse Reactions (AAR) that occur within 60 min after the injection [1]. Although the incidence of AAR is low, serious consequences can still occur, such as hypotensive shock, respiratory arrest, or cardiac arrest, and almost all life-threatening AARs occur within the first 20 min after injection [2]. Therefore, accurately predicting the risk of ICM-related AAR is meaningful for reducing its occurrence by early intervention.

The American College of Radiology reported that the incidence of ICM-related AAR in males is lower than that in females [2]. Motosugi *et al*. not only confirmed the previous viewpoint, but also suggested that youth are more likely to experience ICM-related AAR than the elderly [4]. European Society of Urogenital Radiology pointed out that the presence of allergic disease is a risk factor for ICM-related AAR, and patients with moderate to severe ICM-related AAR should use a non-ionic iodine-based contrast medium [1]. Research by Ha *et al*. and Park *et al*. reported that the injection speed is also an independent prognostic factor for it [5, 6]. The occurrence of ICM-related AAR involves the above factors, which still makes the prediction of ICM-related AAR a challenging task in clinical settings [1-3]. Therefore, there is an urgent need to design a more reliable and accurate comprehensive prediction strategy by integrating various risk factors for early identification of AAR so as to prevent it.

Recently, with the progress of digital biomedical and clinical data, and advances in Machine Learning (ML), the application of ML models in disease risk prediction has surged [7]. ML models commonly extract disease-related features from large amounts of clinical data without relying on hypothetical mechanisms to discover patterns in data; therefore, they excel at training to learn complex relationships between input features and predicted targets, and generate accurate predictions [8]. Common ML includes Logistic Regression (LR), Decision Tree (DT), K-Nearest Neighbors (KNN), and Linear Support Vector Classification (LinearSVC). To our understanding, an ML-based risk model for predicting ICM-related AAR has not been reported. The European Society of Urogenital Radiology has reported that patients with a history of previous ICM-related AAR can be at high risk of recurrent AAR during a second similar contrast-medium administration, and recommended an alternative test not requiring a contrast agent of similar class, or use of a different contrast agent [1]. For majority of patients without a history of ICM-related AAR, this study aimed to comprehensively analyze the influencing factors of ICM-related AAR, establish a predictive model for identification of ICM-related AAR, compare the predictive performance and applicability of different ML models to select the superior model to develop a nomogram, for early identification of AAR high risk groups before ICM injection so as to prevent it in clinical practice.

## 2 METHODS

### 2.1 Ethical Approval

This study has been approved by the Ethics Committee of The Second Affiliated Hospital of Chongqing Medical University in China with the approval number (2022) 728, September 19, 2023, and conducted in compliance with the principles of the Declaration of Helsinki. Patients have signed an informed consent form for ICM injection before undergoing a contrast-enhanced CT examination.

### 2.2 Study Participants

The patients undergoing contrast-enhanced CT examinations were collected through the picture archiving and communication system, and an overview of the study population and design is depicted in Fig. (**1**). The inclusion criteria were the following: (1) the patients aged 18 years or older, (2) the patients without a history of previous ICM-related AAR, (3) the patients underwent contrast-enhanced CT scan with ICM, (4) all patients met the indications for CT contrast-enhanced examination, and (5) the individual record was complete. The exclusion criteria were the following: (1) patients with delayed adverse reactions, (2) patients who interrupted the injection during CT scans, or (3) patients with ICM-related AAR mimic symptoms before ICM injection. Between April 2022 and April 2024, we retrospectively collected 247,976 patients from 4 institutions, of which 226 patients with ICM-related AAR were diagnosed. AAR was diagnosed by an onsite radiologist based on the current symptoms of chemo-toxic responses such as nausea and vomiting, as well as allergy-like or hypersensitivity reactions (Table **1**), but excluded contrast extravasation that developed within contrast administration [1, 2, 9]. Among the 226 patients, 139 patients were from Center 1, 31 patients were from Center 2, 25 patients were from Center 3, and 31 patients were from Center 4.

In addition, we aimed to determine a sample size that provided sufficient statistical power to detect clinically meaningful effects. We enrolled 247,976 patients across four institutions to ensure the detection of significant predictive factors for such low-incidence events. To construct and validate our machine learning models, we required an adequate sample size to mitigate overfitting and ensure generalizability. Thus, we randomly selected 320 patients without AAR from the remaining 247,750 individuals and matched them with the 226 AAR patients based on key variables, including patient status (inpatient, outpatient, or emergency), ICM batch number, and examination type. This approach ensured that each subset (training, test, and external validation set) contained sufficient cases to support reliable model training and evaluation. Given the retrospective nature of this study, the sample size was constrained by the availability of historical data. Ultimately, we included 546 eligible patients from the four centers. This sample size was determined to be adequate to meet the statistical requirements of the study while maintaining practical feasibility.

Among the 226 patients with ICM-related AAR, 176 had mild AAR, and the remaining 50 had moderate to severe AAR. The severe ICM-related AAR included hypotensive shock, respiratory arrest, cardiac arrest, and arrhythmia convulsion [1]. In order to promptly manage ICM-related AAR, we required patients to be monitored in the waiting room for half an hour after injecting ICM [1]. When the patients left, we instructed them to be monitored for half an hour later. In detail, the hospitalized patients were monitored by the clinicians in the ward, while the outpatients were monitored by themselves after leaving the medical environment. They would return to the radiology department for further treatment if there was any discomfort. When AAR occurred, patients with mild AAR were asked to alleviate the symptoms by oral rehydration to relieve dizziness, nausea, and other symptoms, because oral hydration promoted the excretion of contrast agents from the body to reduce blood drug concentration. For patients with moderate to severe AAR, the physicians at the emergency department were immediately notified for symptomatic treatment [10]. In all the 226 patients with ICM-related AAR, 225 patients recovered after the previous intervention, except for one deceased patient due to severe myocardial infarction and AAR of laryngeal edema. They were all recorded in the picture archiving and communication system and the China hospital pharmacovigilance system of the national center for adverse drug reactions monitoring (https://chps.adrs.org.cn/ chpsm/jsp/login.jsp). From the remaining 247,750 patients without AAR, we randomly selected 320 patients to match the 226 with AAR according to the statuses of patients (inpatient/outpatient/emergency department), the batch number of ICM, and the examination items to develop the subsequent models. The data from Center 1 were randomly divided into a training set (70%) and a testing set (30%), with patients from the other three centers serving as the external validation set.

### 2.3 Data Collection

Before the contrast-enhanced CT scans, an ICM (Iopamidol, Iopromide, Ioversol, Iohexol, Iobitridol, or Iomeprol) was injected into a vein of patients *via* an 18- to 20-G needle, followed by 20 mL saline flush *via* a pump injector. Based on previous research and clinical experience [1, 2, 4-6, 11-17], we collected 15 potential predictive factors related to ICM-related AAR, including patient demographics, medical information, laboratory test results, and environmental factors. The patient demographics were gender, age, history of ICM exposure and allergy due to other factors, hypertension degrees, hyperlipidemia, metformin use within 48 h prior to CT scan, and oral hydration amount within 4 h prior to CT scan. Among them, hyperlipidemia was diagnosed according to one or more of the following fasting venous plasma test indicators: (1) total cholesterol ≥ 6.2 mmol/L, (2) low-density lipoprotein cholesterol ≥ 4.1 mmol/L, (3) triglycerides ≥ 2.3 mmol/L, and (4) high-density lipoprotein cholesterol is less than 1.0 mol/L. Medical information was the type of ICMs, ICM material, dose, and rate of ICM injection. The dose and rate of ICM injection were usually determined on the examination items and the patient’s weight, which were 50-85 ml and 3.0-5.0 ml/s, respectively. We also recorded the laboratory test results within 7 days prior to the CT scan: concentration of blood creatinine and Glomerular Filtration Rate (GFR). The normal range of concentration of blood creatinine was 54-133 mmol/L for males and 44-97 mmol/L for females. The normal GFR was more than 100 ml/min, which was derived from the Chronic Kidney Disease Epidemiology Collaboration formula. In addition, we recorded the seasons on environmental factors.

### 2.4 Factor Filtration and Model Building

Univariate analysis of variance was conducted on all potential clinical risk factors, and variables with a *p* value < 0.05 entered the next step. In order to screen for the most valuable predictive factors for predicting ICM-related AAR, the Least Absolute Shrinkage and Selection Operator (LASSO) LR model, which could perform high-dimensional data regression, was applied in the training set. It regularized the regression coefficients by applying L1 penalties. L1 penalty forced the coefficients of the least significant variables to be zero, so these variables were eliminated from the model. The ML model was implemented using R software version 4.2.1, with the input being the dataset required for training and the algorithm, and the output being the predicted results of whether ICM-related AAR occurred. The data were standardized during modeling. Regression variables were trained using four popular and representative ML algorithms, including LR, DT, KNN, and LinearSVC. LR is implemented as a linear model for classification, and the probabilities describing the possible outcomes of a single trial are modeled using a logistic function. DT is a non-parametric supervised learning method used for classification and regression, and its goal is to create a model that predicts the value of a target variable by learning simple decision rules inferred from the data features. KNN is a simple but highly effective classification and regression algorithm, whose core idea is that if the majority of the K nearest samples in the feature space belong to a certain category, then the sample also belongs to that category. LinearSVC is similar to SVC with parameter kernel = 'linear'; it has more flexibility in the choice of penalties and loss functions, and should scale better to large numbers of samples. At the same time, this class supports both dense and sparse input, and the multiclass support is handled according to a one-vs-the-rest scheme. Each of the four ML algorithms has its advantages. LR has the strongest interpretability, the ability to output risk probabilities, and high stability. DT has an intuitive structure and can generate decision tree diagrams, KNN is insensitive to noisy data and does not require complex training, and LinearSVC has strong processing capability for high-dimensional data and supports sparse input. Each model was optimized by 5 repeats of 10-fold cross-validation or tuned to the best parameters. Then, a nomogram was established as a tool for clinical application.

### 2.5 Statistical Analysis

All statistical analyses were undertaken using R software version 4.2.1. Significance was set at a *p*-value < 0.05. The baseline characteristics and outcomes of the study population in each research set were calculated. Continuous variables were represented as median [interquartile range: 25th to 75th percentile], while categorical variables were represented as numbers (percentages). We built each model in the training set and evaluated the performance in the test and validation sets. Each model's performance in the three sets was then assessed using the Area Under the Receiver Operating Characteristic (ROC) Curve (AUC), sensitivity, specificity, accuracy, and F1-score. Model calibration was measured using the calibration curve and DeLong's test. A nomogram was drawn based on the best model’s performance.

## 3 RESULTS

### 3.1 Baseline Characteristics of Patients for Developing Models

Among the 247,976 patients who received intravenous ICM during contrast-enhanced CT scan in four centers, the overall AAR incidence was 0.091% (226 of 247,976), among which 22.12% (50 of 226) had moderate to serious AAR. In order to develop models for AAR prediction, a total of 546 eligible participants, including the previous 226 with AAR and 320 without AAR, were obtained as the final samples, and their baseline characteristics are detailed in Table **2**. There were significant differences in gender, history of ICM exposure and allergy due to other factors, kind of ICM, ICM dose, hypertension degree, oral metformin 48 h prior to CT, hyperlipidemia, and GFR between groups with and without ICM-related AAR (all *p*-values < 0.05). We considered a split-by-random study design; the total cohort from Center 1 (n = 335) was randomly divided into two data sets: 70% in the training set (n = 234) and 30% in the test set (n = 101). We also collected patients from the other three centers as the external validation set (n = 211). There was no statistical difference in data distribution among the three sets (all *p*-values > 0.05).

### 3.2 Predicting Variables for ML Models

In the training set, we explored the potential risk factors for ICM-related AAR in patients. Using univariate analysis, nine potential risk factors associated with ICM-related AAR were preliminarily screened out, including gender, history of ICM exposure and allergy due to other factors, kind of ICM, ICM dose, hypertension degree, oral metformin 48 h prior to CT, hyperlipidemia, and GFR (all *p*-values < 0.05, Table **3**). Further LASSO regression analysis showed that eight variables, including history of ICM exposure and allergy due to other factors, kind of ICM, ICM dose, hypertension degree, oral metformin 48 h prior to CT, hyperlipidemia, and GFR, had significant correlations with ICM-related AAR (all *p*-values < 0.05, Table **3**). Ultimately, the above eight variables were used for the subsequent establishment of ML models.

### 3.3 Establishment and Comparison of Models

**Fig. (2):**
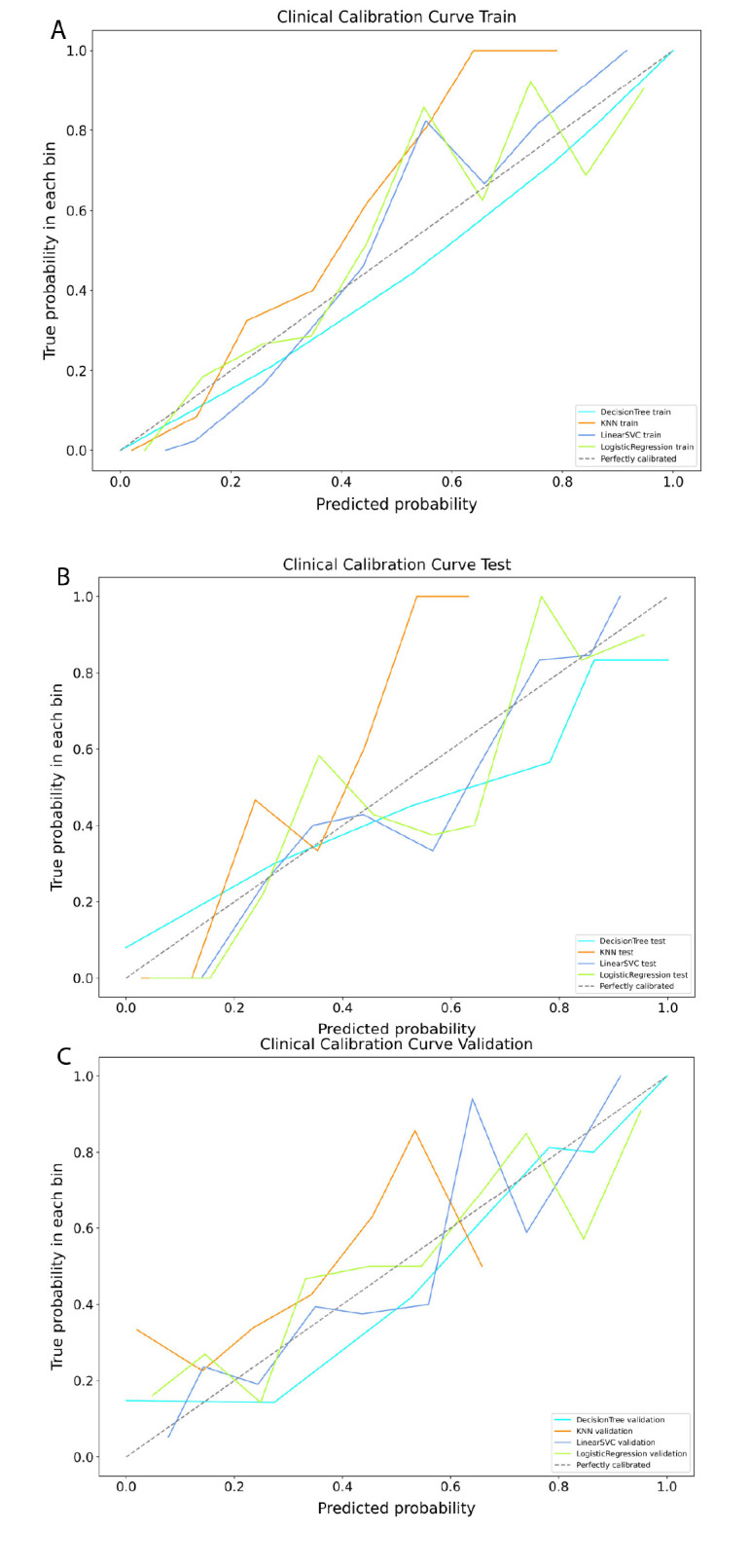
Clinical calibration curve validation in the training, test and validation sets. Images **A**, **B** and **C** show the calibration curve of models in the training, test and validation sets, respectively. Notes: KNN, k-nearest neighbors; LinearSVC, linear support vector classification.

Using the above eight variables with statistical significance, we established four ML models, including LR, DT, KNN, and LinearSVC for predicting ICM-related AAR. The confusion matrices of four models (Figs. **S1**-**S3**) show that the true positive and true negative account for the majority of the dataset, whether in the training set, or in the test and validation sets. The sensitivity, specificity, accuracy, and F1 score of the four prediction models are shown in Table **4**. LR had the highest predictive performance in the test set. Meanwhile, LR demonstrated high efficiency in the external validation set. In addition, calibration curves were used to reflect the match between the predicted probabilities and the actual results (Fig. **2**). The combined under-sampling and 10-fold cross-validation procedures were repeated ten times, and averages were used to estimate the AUC. The test AUC score ranged from 0.768 to 0.894, with LR being the highest (95% confidence intervals (CI), 0.835 to 0.953), followed by LinearSVC and KNN, and DT being lower.

The AUCs of the four ML models in the external validation set are shown in Fig. (**3**). The AUC score in the external validation ranged from 0.664 to 0.814, with LR being the highest (95% CI, 0.753 to 0.874), followed by LinearSVC, DT, and KNN being lower. The DeLong’s test showed that the AUC of the LR model was significantly higher than that of DT, KNN, or LinearSVC model (*p*-values = 0.0009, 0.0268, and 0.0155, respectively), and that the AUC of the DT model was significantly higher than that of the LinearSVC model (*p* = 0.0159). In contrast, there were no significant differences in AUC between DT and KNN models (*p* = 0.5642), and between KNN and LinearSVC models (*p* = 0.204).

### 3.4 Development of an Individualized Nomogram

In order to provide clinicians with a visual tool to ensure easy use of the model for predicting the individual probability of ICM-related AAR patients, we presented the LR model with the highest discrimination performance as a nomogram. The 8 candidate factors after univariate analysis and LASSO feature selection were screened to form the final nomogram, as shown in Fig. (**4**).

## 4 DISCUSSION

The study used LR, DT, KNN, and LinearSVC to build risk prediction models for ICM-related AAR in patients without a history of AAR. After calculating the four ML algorithms and comparing pairwise with DeLong’s test, the LR model performed best based on various metrics, and was subsequently converted into a nomogram for easier use in clinical settings.

This study reveals that 8 variables are combined to build the prediction models for ICM-related AAR for the first time. GFR is a variable associated with ICM-related AAR prediction, because it may be related to drug metabolism, expressed as the half-life rate of ICM [18]. In patients with partial renal impairment, the half-lives of ICM increase progressively [19, 20]. As another predictor of AAR, hyperlipidemia may be related to the physicochemical properties of ICM and blood viscosity. It is generally believed that the release of corresponding active mediators by mast cells and eosinophils is caused by drug components or complement activation, which specifically bind with IgE antibodies, promote the release of inflammatory mediators such as histamine, and ultimately exhibit various allergic symptoms [21]. In fact, the mechanism of ICM-related AAR is quite complex, and there is research barely investigating the potential mechanisms, and there is a lack of consensus [22, 23]. The history of ICM exposure may be related to the release of corresponding active mediators by eosinophils and mast cells [24, 25]. More doses of ICM may cause longer retention in the kidney due to increased concentration and viscosity in the renal tubules after administration [12]. In addition, some studies have shown that types of ICM are related to their side chain structure [26, 27], indicating their different physicochemical properties. Our research indicates that a history of allergic reactions except ICM is a high-risk factor, which is consistent with the published research [6]. Our study also supports the idea that the use of metformin and grade III hypertension are also risk factors, as mentioned in the guidelines [1, 2, 23].

Clinically, we have evaluated multicenter data from four institutions to form a prediction model for ICM-related AAR based on ML for the first time. Our LR model demonstrates the highest performance when compared with the LinearSVC, DT and KNN models in the training (AUC: 88.17% *vs*. 87.96%, 80.72% and 82.65%), test (AUC: 89.39% *vs*. 85.75%, 76.8% and 80.13%) and external validation (AUC: 81.35% *vs*. 79.14%, 79.05% and 66.35%) sets. Subsequently, we have presented the LR model-based nomogram using the above 8 candidate factors to provide a visual tool to predict the risk of ICM-related AAR for clinicians, which may be potentially used for the timely identification of a patient population at high risk before contrast-enhanced CT scan in order to prevent the AAR in advance. If the patients are identified as high risk of ICM-related AAR, they could receive an ICM agent known to cause fewer hypersensitivity reactions, or they should undergo contrast agent switching and the examination should be performed at a site with equipment ready to treat more serious reactions.In addition, ML modeling was used in this study to develop ICM-related AAR prediction models. ML mainly provides a practical technology for artificial intelligence in the big data era, collecting, modeling, and analyzing medical big data accumulated in hospital information systems, and can be used to reasonably predict and determine the known types of unknown data [28]. The massive amount of medical data also provides data support for exploring potential risk factors. As in a previous report [29], we developed and validated an ML nomogram by integrating clinical factors with satisfactory performance in predicting the risk of ICM-related AAR.

## 5 LIMITATIONS

However, there are still several inevitable limitations in this study. Firstly, we kept the patients in a medical environment for 30 minutes after ICM injection according to the guidelines from the European Society of Urogenital Radiology [1], but the patients without AAR left the medical environment after 30 minutes being monitored; they did not return to the department of radiology, indicating that they did not have AAR. Secondly, this study only included patients without a history of ICM allergy. According to the previous guidelines [1], patients with moderate to severe anaphylaxis of ICM were prohibited from contrast-enhanced CT scans, and patients with mild allergy to ICM could receive a similar examination by use of a different contrast agent. Despite this limitation, our findings can be suitable for patients without a history of allergy to ICM. Furthermore, our variable selection was primarily guided by the established risk factors documented in existing guidelines and literature. A valuable direction for future research would be to systematically investigate the effects of a broader spectrum of concomitant medications, including anti-allergic drugs, by collecting more comprehensive drug data.

## CONCLUSION

In summary, this study used ML algorithms to screen out eight clinically easily obtainable risk variables highly correlated with ICM-related AAR, including history of ICM exposure and allergy due to other factors, kind of ICM, ICM dose, hypertension degree, oral metformin 48 h prior to CT, hyperlipidemia, and GFR. The risk prediction model constructed based on the risk variables showed good discrimination and clinical applicability. We constructed a nomogram based on the superior model, which can help clinicians identify high-risk patients before contrast-enhanced CT and take the corresponding measures to prevent the occurrence of ICM-related AAR in advance in clinical settings.

## Figures and Tables

**Fig. (1):**
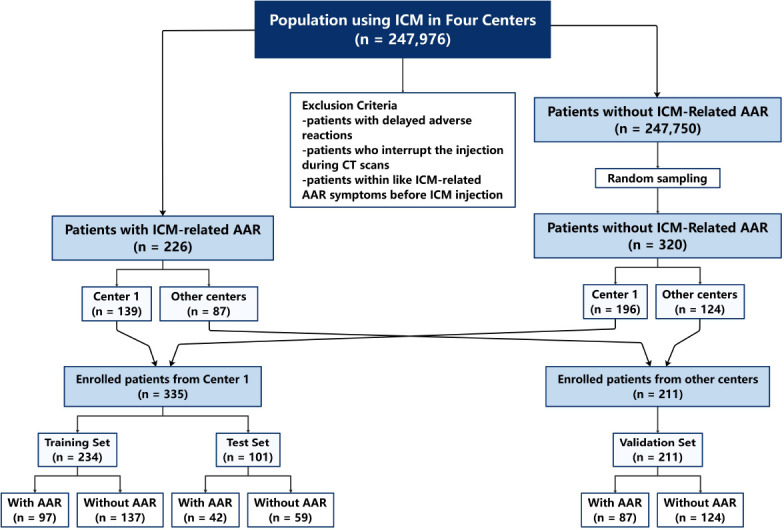
Overview of study population and design. Notes: ICM, iodine contrast media; AAR, acute adverse reaction.

**Fig. (3):**
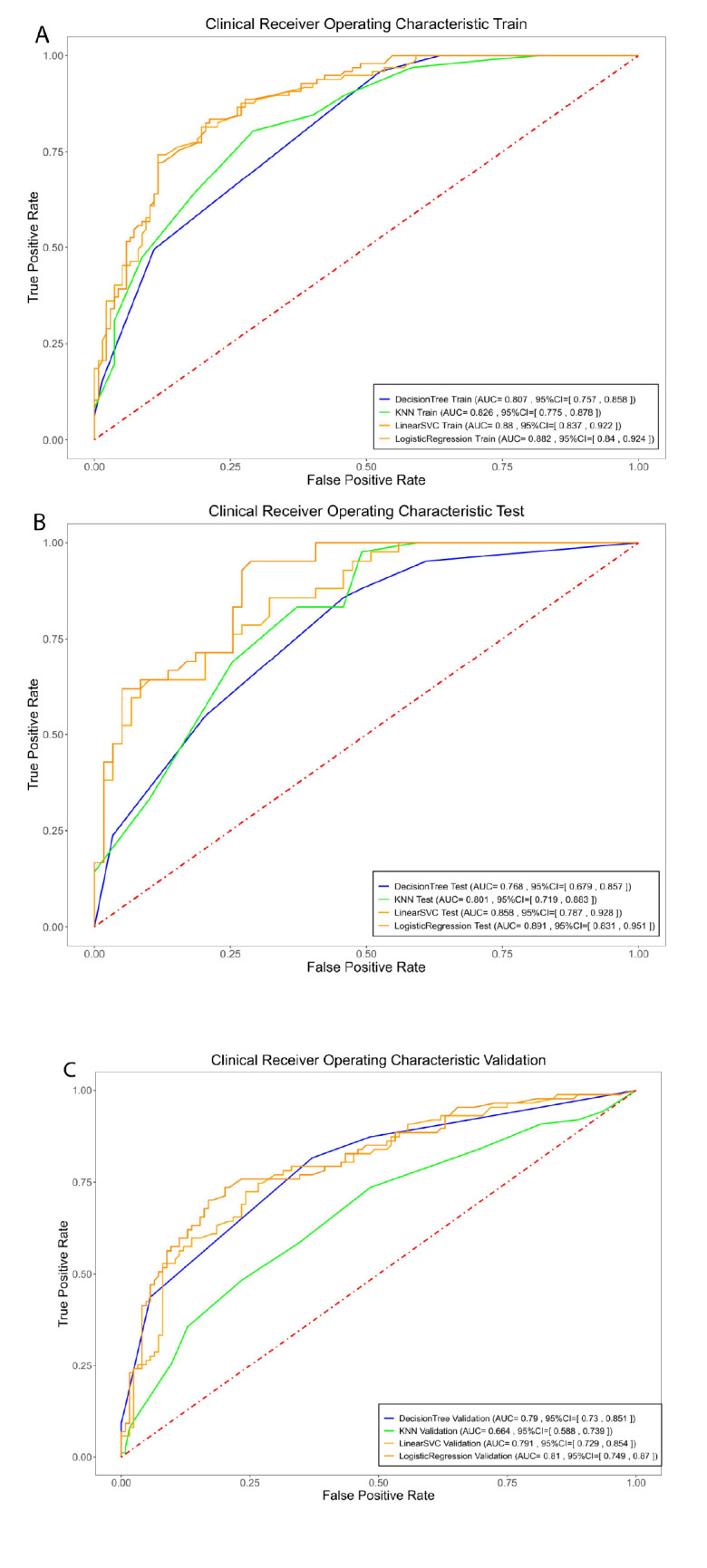
Clinical receiver operating characteristic in the training, test and validation sets. Images **A**, **B** and **C** show the AUC of models in the training, test and validation sets, respectively. Notes: AUC, Area under receiver operating characteristic curve; CI, confidence intervals; KNN, k-nearest neighbors; LinearSVC, linear support vector classification.

**Fig. (4):**
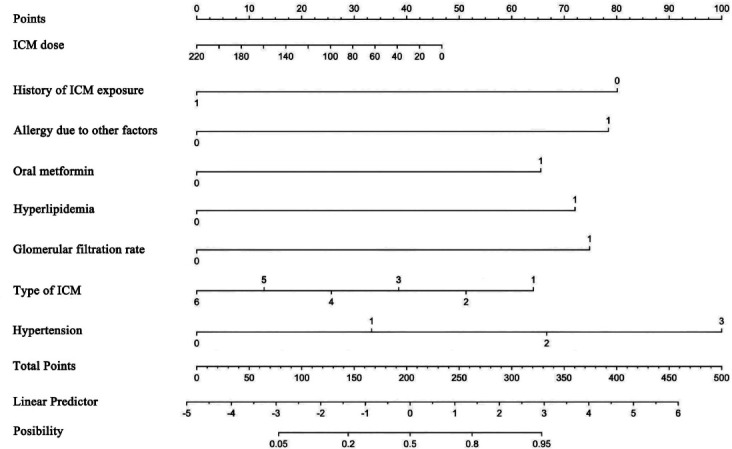
Logistic regression model nomogram. Note: ICM, iodine contrast media.

**Table 1:** Classification of acute adverse reactions [9].

-	Hypersensitivity/Allergy-like	Grade (Ring and Messmer classification)	Chemotoxic
Mild	Mild urticaria	Grade 1	Nausea/mild vomiting
-	Mild itching	Grade 1	Warmth/chills
-	Erythema	Grade 1	Anxiety
-	-	-	Vasovagal reaction which resolves spontaneously
Moderate	Marked urticaria	Marked urticaria	Vasovagal reaction
-	Mild bronchospasm	Grade 2	-
-	Facial/Iaryngeal edema	Grade 2	-
Moderate	Marked urticaria	Grade 1	Vasovagal reaction
Severe	Hypotensive shock	Grade 3	Arrythmia
-	Respiratory arrest	Grade 3	Convulsion
-	Cardiac arrest	Grade 4	-

**Table 2:** Baseline data of patients for developing models.

Variable	Training Set (n = 234)		Test Set (n = 101)		Validation Set (n = 211)	
	With AAR (n = 97)	Without AAR (n = 137)	With AAR (n = 42)	Without AAR (n = 59)	With AAR (n = 87)	Without AAR (n = 124)
Gender (%)	-	-	-	-	-	-
Female	45 (46.4%)	51 (37.2%)	20 (47.6%)	27 (45.8%)	49 (56.3%)	53 (42.7%)
Male	52 (53.6%)	86 (62.8%)	22 (52.4%)	32 (54.2%)	38 (43.7%)	71 (52.3%)
Age (median ± IQR, year)	56 (49 - 68)	57 (49 - 68)	55.5 (50.5 - 64.25)	58 (48.5 - 67.5)	55 (49 - 67)	56 (49 - 68)
History of ICM exposure (%)	-	-	-	-	-	-
No	72 (74.2%)	54 (39.4%)	33 (78.6%)	21 (35.6%)	62 (71.3%)	44 (35.5%)
Yes	25 (25.8%)	83 (60.6%)	9 (21.4%)	38 (64.4%)	25 (28.7%)	80 (64.5%)
Allergies (%)	-	-	-	-	-	-
No	72 (74.2%)	131 (95.6%)	28 (66.7%)	57 (96.6%)	60 (69%)	120 (96.8%)
Allergy due to other factors	25 (25.8%)	6 (4.4%)	14 (33.3%)	2 (3.4%)	27 (31%)	4 (3.2%)
Type of ICM (%)	-	-	-	-	-	-
Iopamidol	22 (22.6%)	16 (11.7%)	9 (21.4%)	14 (23.7%)	19 (21.8%)	25 (20.2%)
Iopromide	44 (45.4%)	46 (33.6%)	18 (42.9%)	15 (25.4%)	24 (27.6%)	39 (31.4%)
Ioversol	11 (11.3%)	23 (16.8%)	9 (21.4%)	10 (17%)	22 (25.3%)	29 (23.4%)
Iohexol	5 (5.2%)	9 (6.6%)	0 (0%)	5 (8.5%)	9 (10.4%)	11 (8.9%)
Iobitridol	6 (6.2%)	17 (12.4%)	0 (0%)	1 (1.7%)	3 (3.4%)	4 (3.2%)
Iomeprol	9 (9.3%)	26 (18.9%)	6 (14.3%)	14 (23.7%)	10 (11.5%)	16 (12.9%)
ICM (mg I/ml)	-	-	-	-	-	-
300	5 (5.2%)	9 (6.6%)	0 (0%)	5 (8.5%)	9 (10.4%)	11 (8.9%)
350	22 (22.6%)	40 (29.2%)	10 (23.8%)	11 (18.6%)	25 (28.7%)	33 (26.6%)
370	60 (61.9%)	62 (45.3%)	1 (2.3%)	29 (49.2%)	43 (49.4%)	64 (51.6%)
400	10 (10.3%)	26 (18.9%)	5 (11.9%)	14 (23.7%)	10 (11.5%)	16 (12.9%)
ICM dose (ml)	80 (80 - 85)	80 (75 - 80)	80 (75 - 85)	80 (75 - 80)	80 (70 - 100)	80 (70 - 100)
ICM injection rate (ml/s)	3 (3 - 3.5)	3 (3 - 3)	3 (3 - 3.5)	3 (3 - 4.25)	3.5 (3 - 4)	3 (3 - 4)
Hypertension (%)	-	-	-	-	-	-
No	45 (46.3%)	92 (67.1%)	14 (33.3%)	33 (55.9%)	57 (65.5%)	81 (65.3%)
Grade 1	34 (35.1%)	41 (29.9%)	22 (52.4%)	20 (33.9%)	19 (21.8%)	32 (25.8%)
Grade 2	9 (9.3%)	2 (1.5%)	1 (2.4%)	5 (8.5%)	7 (8.1%)	10 (8.1%)
Grade 3	9 (9.3%)	2 (1.5%)	5 (11.9%)	1 (1.7%)	4 (4.6%)	1 (0.8%)
Oral metformin 48 h before CT (%)	-	-	-	-	-	-
No	82 (84.5%)	127 (92.7%)	31 (73.8%)	57 (96.6%)	73 (83.9%)	121 (97.6%)
Yes	15 (15.5%)	10 (7.3%)	11 (26.2%)	2 (3.4%)	14 (16.1%)	3 (2.4%)
Hyperlipidemia (%)	-	-	-	-	-	-
No	60 (61.9%)	124 (90.5%)	26 (61.9%)	49 (83%)	59 (67.8%)	118 (95.2%)
Yes	37 (38.1%)	13 (9.5%)	16 (38.1%)	10 (17%)	28 (32.2%)	6 (4.8%)
Creatinine (%)	-	-	-	-	-	-
Normal	81 (83.5%)	122 (89.1%)	38 (90.5%)	53 (89.8%)	74 (85.1%)	104 (83.9%)
Abnormal	16 (16.5%)	15 (10.9%)	4 (9.5%)	6 (10.2%)	13 (14.9%)	20 (16.1%)
GFR (%)	-	-	-	-	-	-
Normal	58 (59.8%)	120 (87.6%)	24 (57.2%)	54 (91.5%)	61 (70.1%)	107 (86.3%)
Abnormal	39 (40.2%)	17 (12.4%)	18 (42.8%)	5 (8.5%)	26 (29.9%)	17 (13.7%)
Hydration amount (ml)	1000 (500 - 1500)	850 (300 - 1500)	1000 (500 - 1250)	600 (300 - 1300)	500 (500 - 600)	500 (500 - 800)
Season (%)	-	-	-	-	-	-
Spring	28 (28.9%)	43 (31.4%)	17 (40.5%)	19 (32.2%)	31 (35.6%)	24 (19.3%)
Summer	34 (35.1%)	50 (36.5%)	10 (23.8%)	21 (35.6%)	30 (34.5%)	78 (62.9%)
Autumn	26 (26.7%)	36 (26.3%)	12 (28.6%)	14 (23.7%)	17 (19.5%)	11 (8.9%)
Winter	9 (9.3%)	8 (5.8%)	3 (7.1%)	5 (8.5%)	9 (10.4%)	11 (8.9%)

**Notes: **IQR, interquartile range; ICM, iodine contrast media; AAR, acute adverse reactions; GFR, global filtration rate.

**Table 3:** Univariate analysis and LASSO of screening predictive factors in the training set.

Variable	Univariate Analysis *p*-value	LASSO Coefficient
Gender	0.16	-
Age	0.644	-
History of ICM exposure	**<0.01**	-1.224033992
Allergies	**<0.01**	0.8838854564
Type of ICM	-	-
Iopamidol	**0.025**	0.4694896475
Iopromide	0.068	0.1512254160
Ioversol	0.244	0.0137635585
Iohexol	0.653	-0.077074749
Iobitridol	0.115	-0.257051697
Iomeprol	**0.040**	-0.351100303
ICM (mg I/ml)	0.076	-
ICM dose	**0.011**	0.0024517603
ICM injection rate	0.055	-
Hypertension	**<0.01**	-
No	-	-0.65951137
Grade 1	-	-0.117406148
Grade 2	-	0.3344281660
Grade 3	-	0.3754706652
Oral metformin 48 h before CT	**0.046**	0.4747935805
Hyperlipidemia	**<0.01**	1.0563134180
Creatinine	0.218	-
GFR	**<0.01**	1.0725759884
Hydration amount	0.904	-
Season	-	-
Spring	0.679	-
Summer	0.820	-
Autumn	0.928	-
Winter	0.318	-

**Notes: **LASSO, least absolute shrinkage and selection operator; IQR, interquartile range; ICM, iodine contrast media; GFR, global filtration rate. The bold entries represent the statistically significant differences between patients with and without ICM-related AAR.

**Table 4:** Predictive performance of the four machine learning models.

Method	Data Set	AUC	Sensitivity	Specificity	Accuracy	F1 Score
LR	Training	0.8817	0.8351	0.7883	0.8077	0.7826
	Test	0.8939	0.881	0.7458	0.802	0.7872
	Validation	0.8135	0.7011	0.8306	0.7773	0.7219
DT	Training	0.8072	0.9588	0.4745	0.6752	0.7099
	Test	0.768	0.881	0.5085	0.6634	0.6852
	Validation	0.7905	0.8736	0.5161	0.6635	0.6816
KNN	Training	0.8265	0.8041	0.708	0.7479	0.7256
	Test	0.8013	0.8333	0.6271	0.7129	0.7071
	Validation	0.6635	0.4828	0.7661	0.6493	0.5316
LinearSVC	Training	0.8796	0.7423	0.8832	0.8248	0.7784
	Test	0.8575	0.7381	0.7458	0.7426	0.7045
	Validation	0.7914	0.5977	0.8629	0.7536	0.6667

**Notes: **AUC, area under receiver operating characteristic curve; LR, logistic regression; DT, decision tree; KNN, k-nearst neighbors; LinearSVC, linear support vector classification.

## Data Availability

The relevant data and materials can be obtained from the corresponding author [X.W] on reasonable request.

## LIST OF ABBREVIATIONS

ICM: Iodine Contrast Media
AAR: Acute Adverse Reactions
LASSO: Least Absolute Shrinkage and Selection Operator
ML: Machine Learning
LR: Logistic Regression
DT: Decision Tree
KNN: K-Nearest Neighbors
LinearSVC: Linear Support Vector Classification
ROC: Receiver Operator Characteristic
AUC: Area Under the Receiver Operating Characteristic Curve
CI: Confidence Intervals
GFR: Glomerular Filtration Rate

## STANDARDS OF REPORTING

STROBE guidelines were followed.
