# Supplementary material for: Machine Learning Model to Predict Iodine Contrast Media-related Acute Adverse Reaction in Patients without a Similar History for Enhanced CT
Source: Curr Med Imaging. 2025 Oct 27;21:e15734056436322. doi: 10.2174/0115734056436322251022040623 (PMC13137357; doi:10.2174/0115734056436322251022040623)
Supplement: Supplementary file 1 [file CMIM-21-E15734056436322_SD1.pdf]

1573-4056/25 *Send Orders for Reprints to reprints@benthamscience.net*

BENTHAM SCIENCE

CrossMark

Current Medical Imaging

Content list available at: https://benthamscience.com/journals/cmir

Supplementary Material

Machine Learning Model to Predict Iodine Contrast Media-related Acute Adverse Reaction in Patients without a Similar History for Enhanced CT

Ke-xin Jiang[1], Wen-yan Liu[1], Yang Xu[1], Kun-hua Li[1], Fang Wen[2], Rong Zhou[3], Shi-lan Xiang[4], Da-jing Guo[1], Tian-wu Chen[1] and Xiao-lin Wang[1,*]

[1]*Department of Radiology, The Second Affiliated Hospital of Chongqing Medical University, No.74 Linjiang Road, Yuzhong District, Chongqing 400010, China*
[2]*Department of Radiology, Chongqing University Three Gorges Hospital, 165# Xincheng Street, Wanzhou District, Chongqing 404100, China*
[3]*Department of Radiology, Yongchuan Hospital of Chongqing Medical University, 439# Xuanhua Street, Yongchuan District, Chongqing 402160, China*
[4]*Department of Radiology, Chongqing University Qianjiang Hospital, 360# Zhengzhou South Street, Qianjiang District, Chongqing 409000, China*

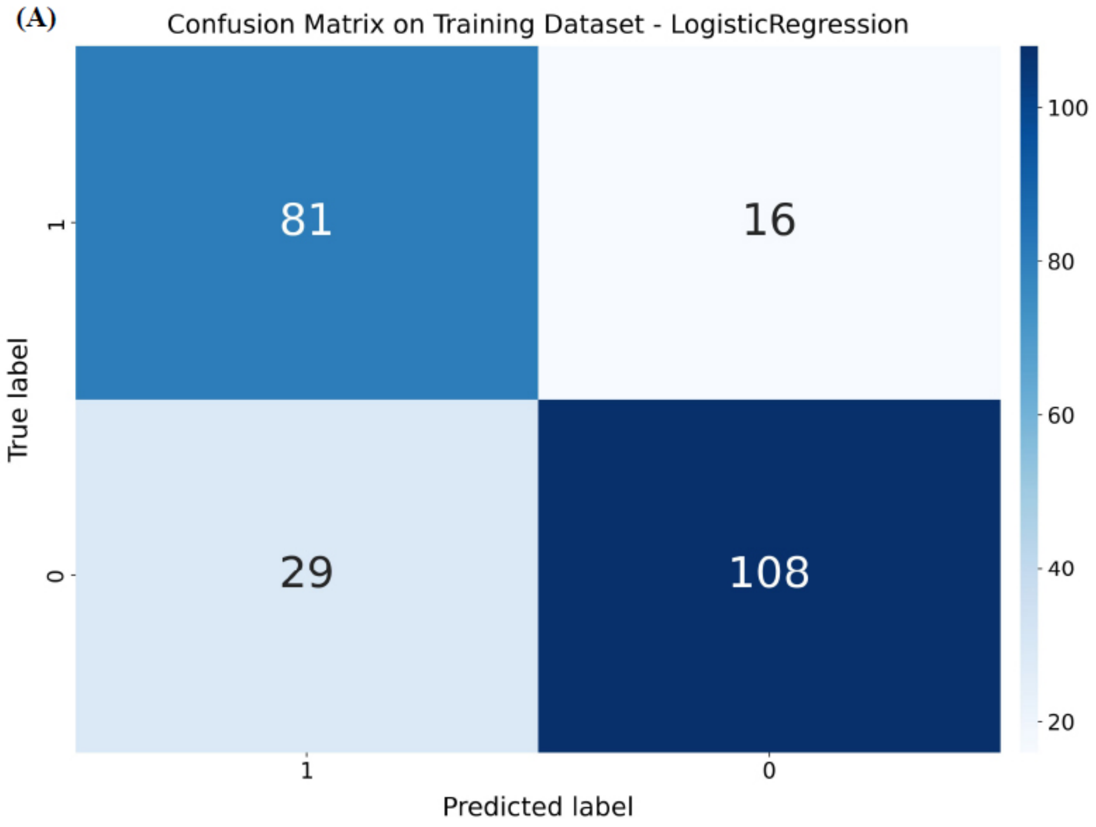

Fig. S1 contd.....

DOI: 10.2174/0115734056436322251022040623, 2025, *21*, e15734056436322

(B)

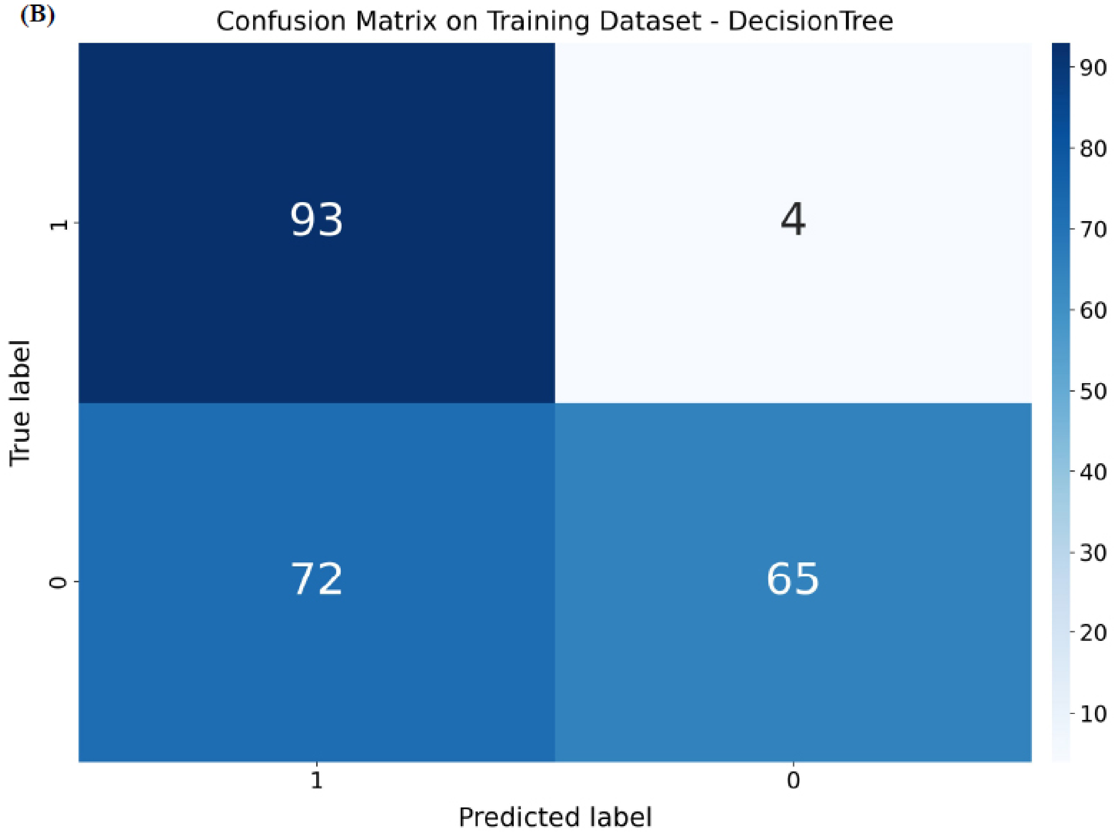

(C)

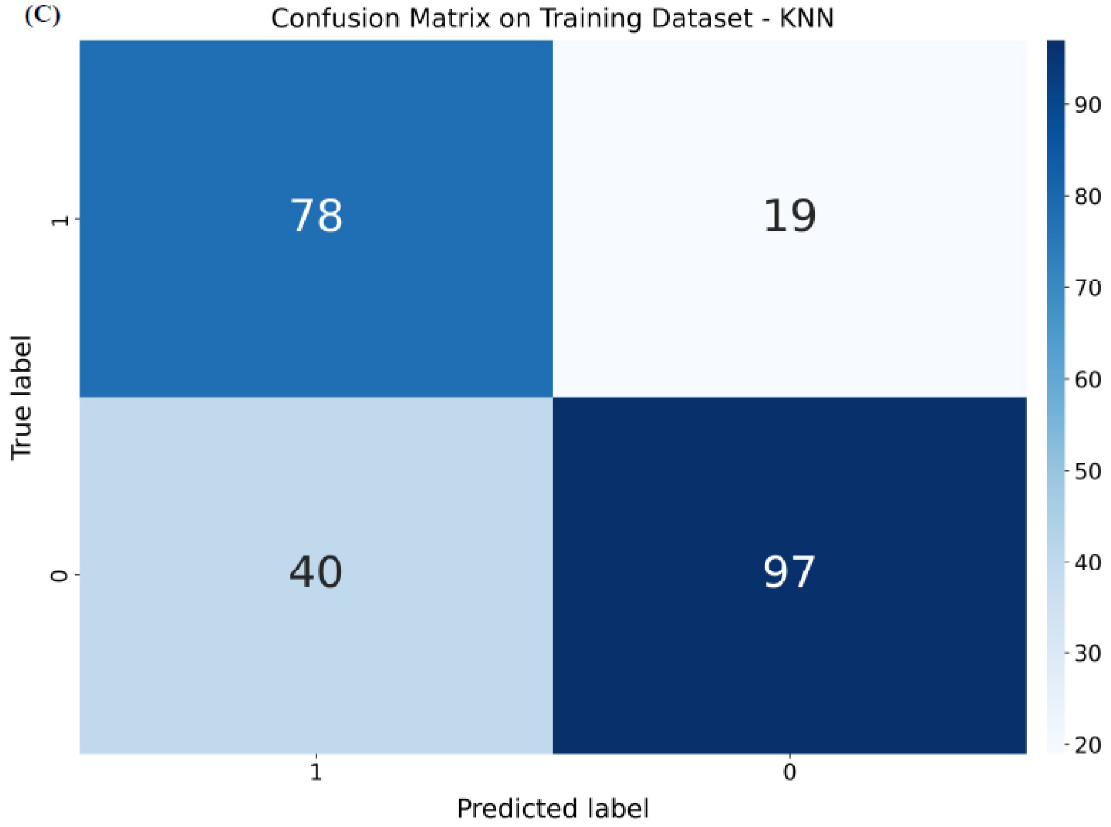

Fig. S1 contd.....

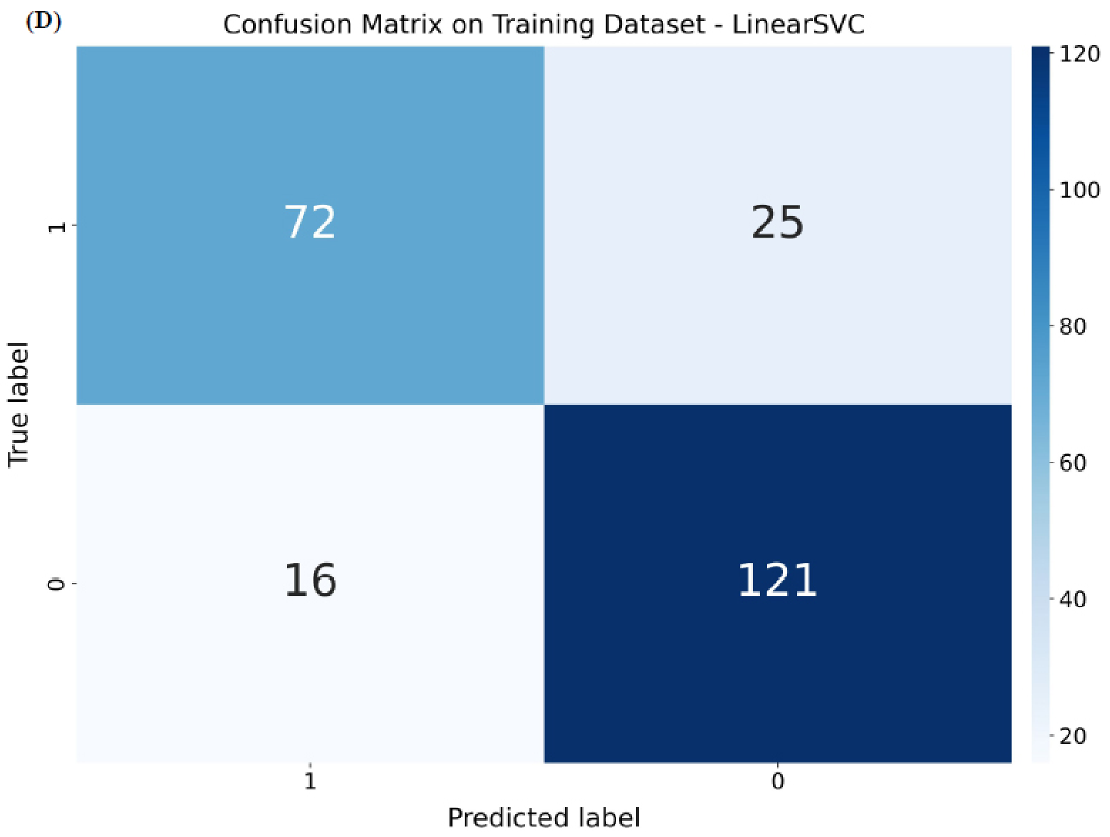

Fig. (S1). The Confusion Matrix of logistic regression (**A**), decision tree (**B**), KNN (**C**) and LinearSVC (**D**) in the training set, respectively. Notes: KNN, k-nearest neighbors; LinearSVC, linear support vector classification.

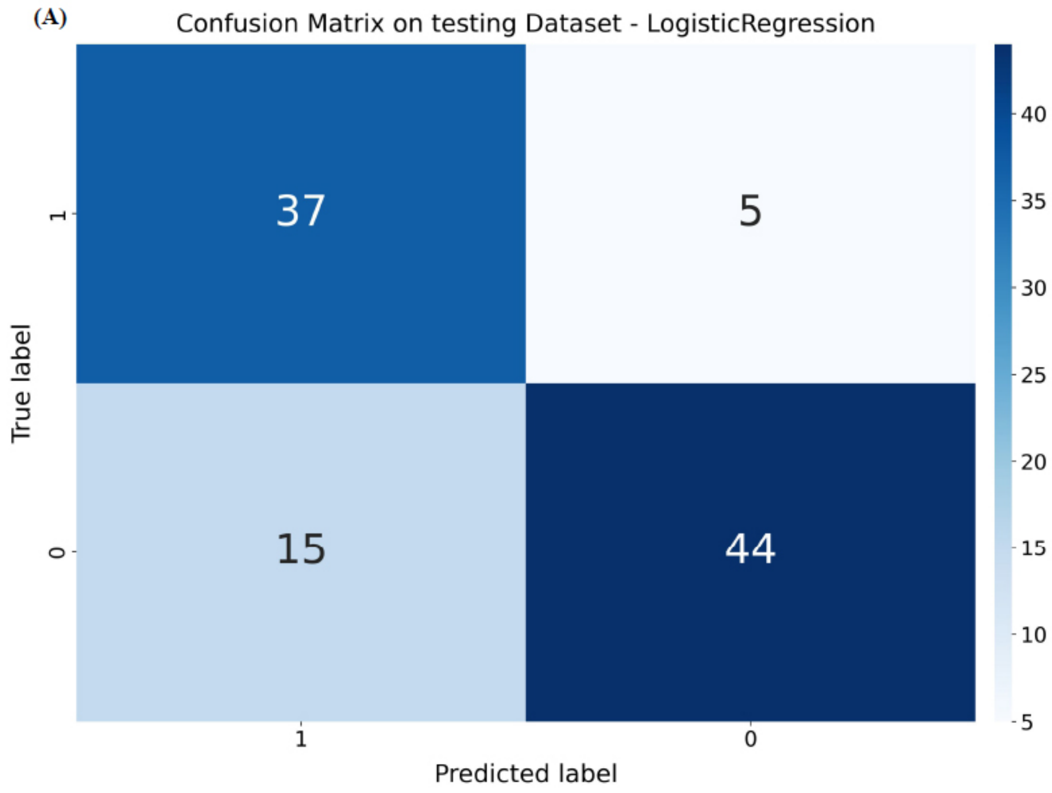

Fig. S2 contd.....

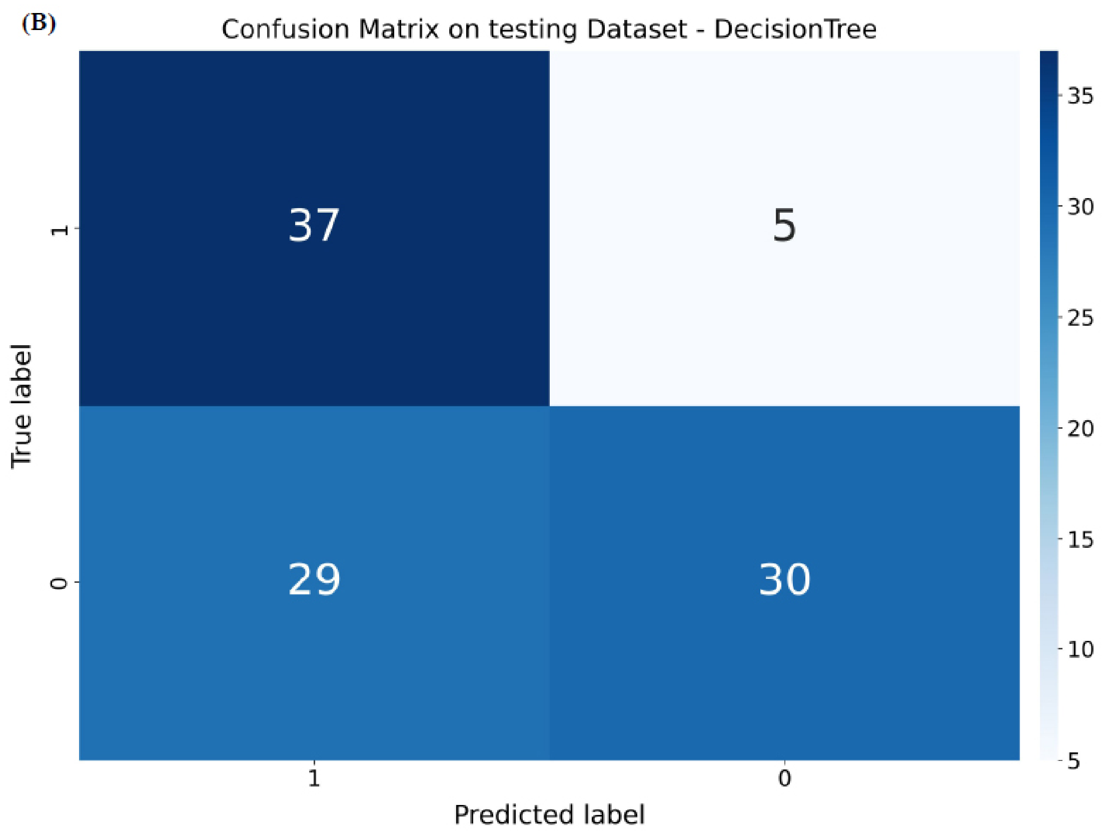
(B)
Confusion Matrix on testing Dataset - DecisionTree
37
5
29
30
True label
1
0
Predicted label
35
30
25
20
15
10
5

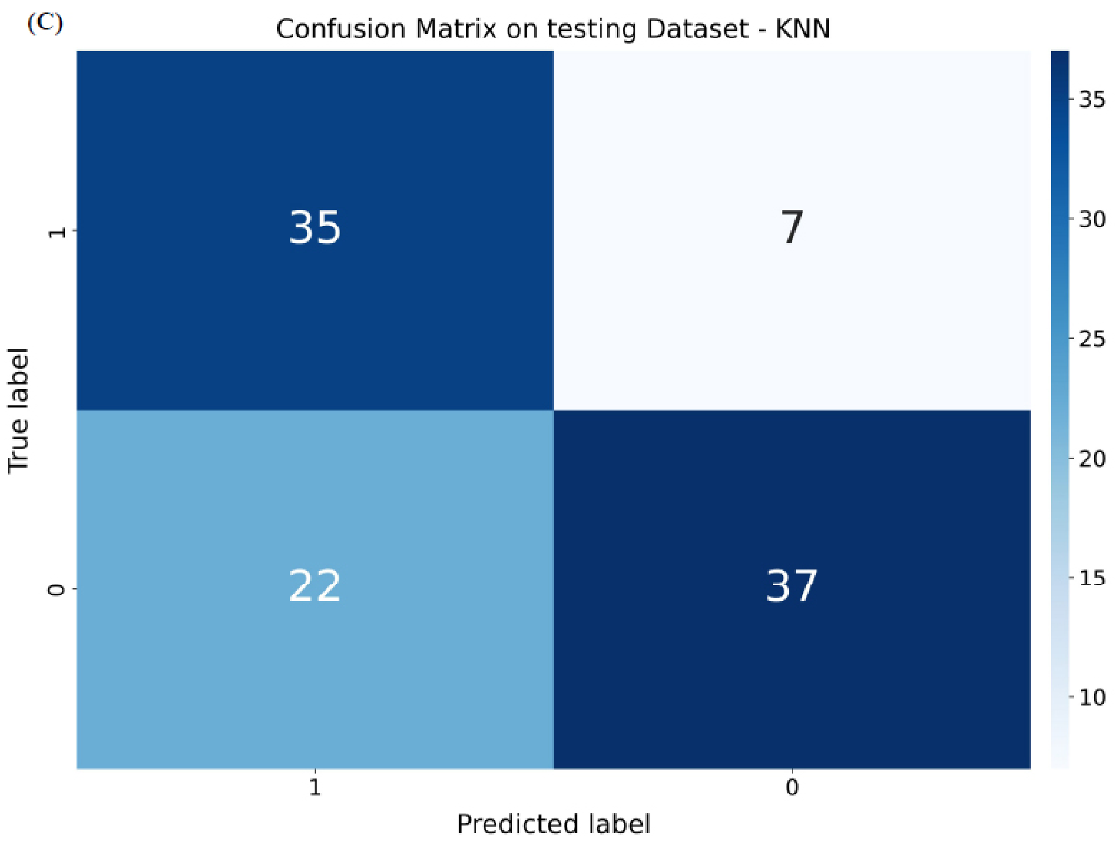
(C)
Confusion Matrix on testing Dataset - KNN
35
7
22
37
True label
1
0
Predicted label
35
30
25
20
15
10

Fig. S2 contd.....

(D)

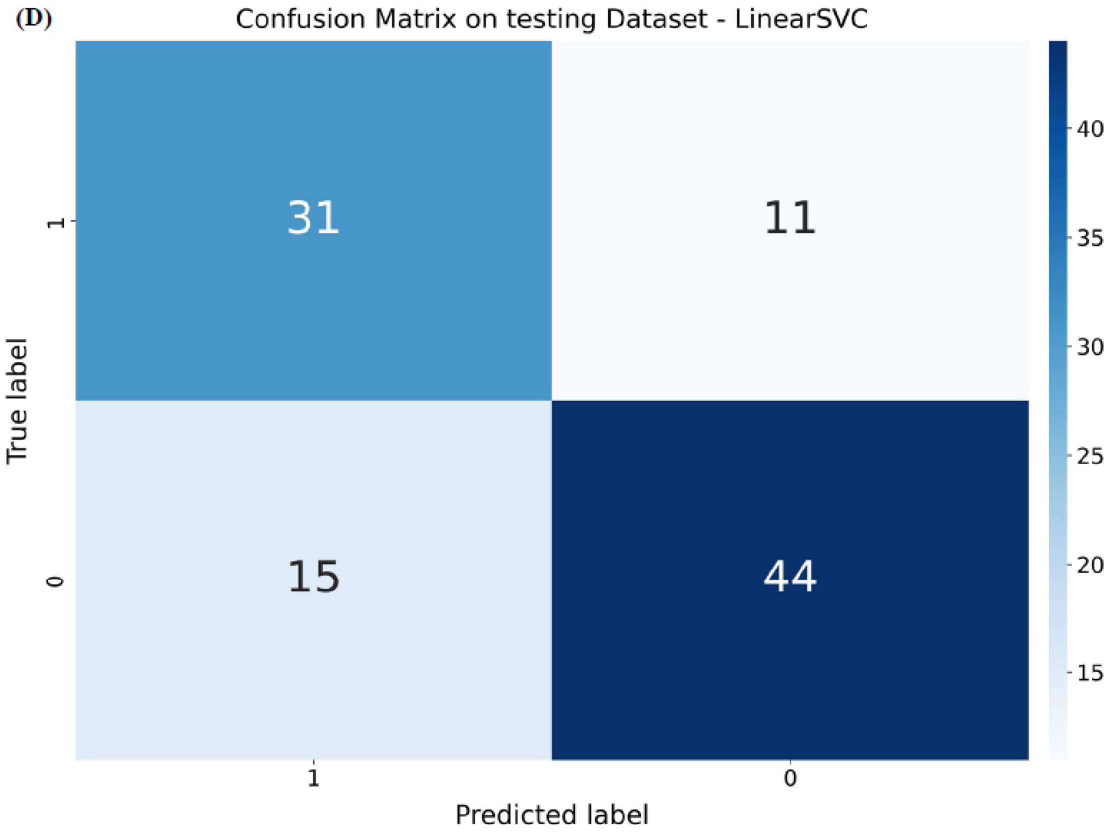

Fig. (S2). The Confusion Matrix of logistic regression (**A**), decision tree (**B**), KNN (**C**) and LinearSVC (**D**) in the test set, respectively.
Notes: KNN, k-nearest neighbors; LinearSVC, linear support vector classification.

(A)

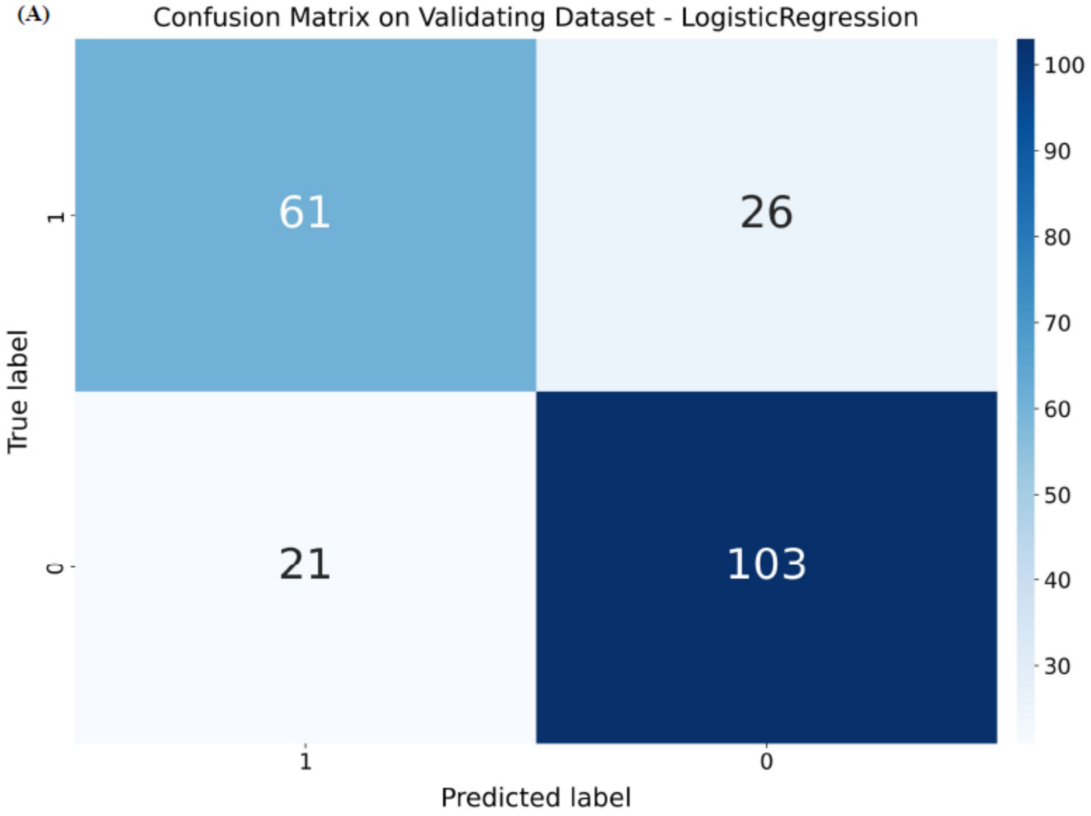

Fig. S3 contd.....

(B)

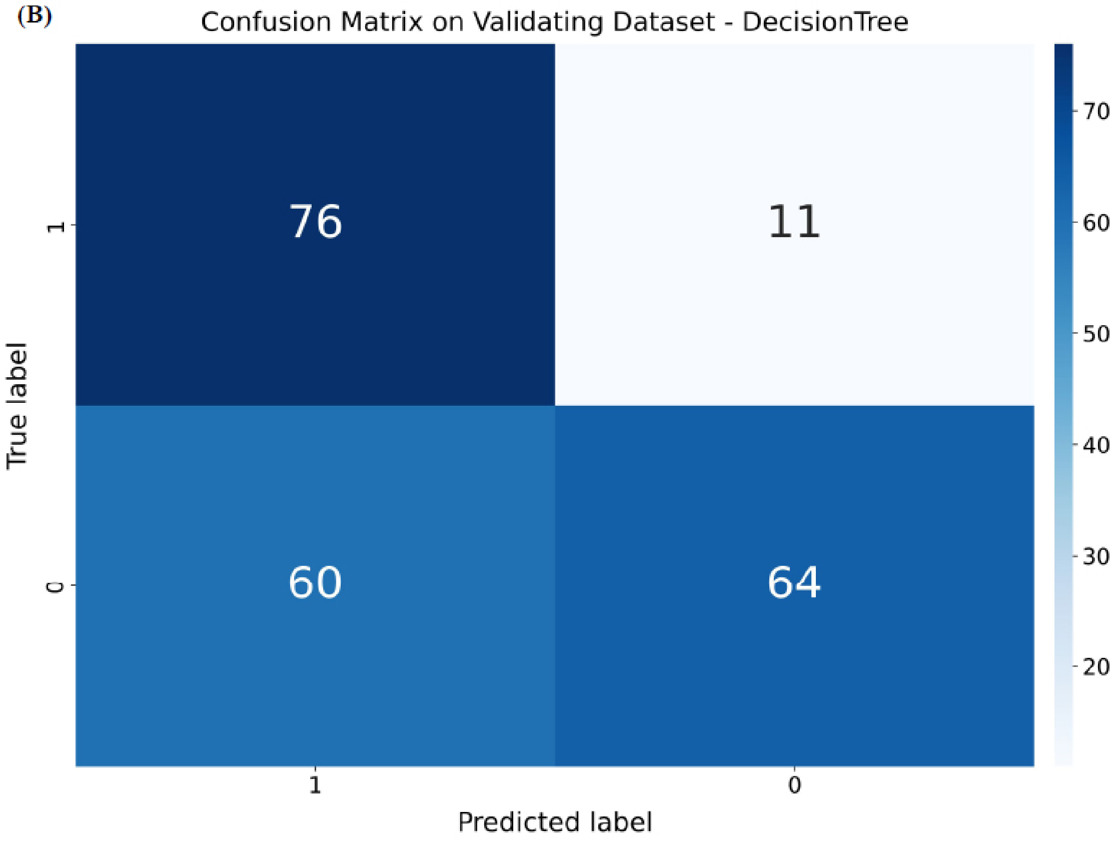

(C)

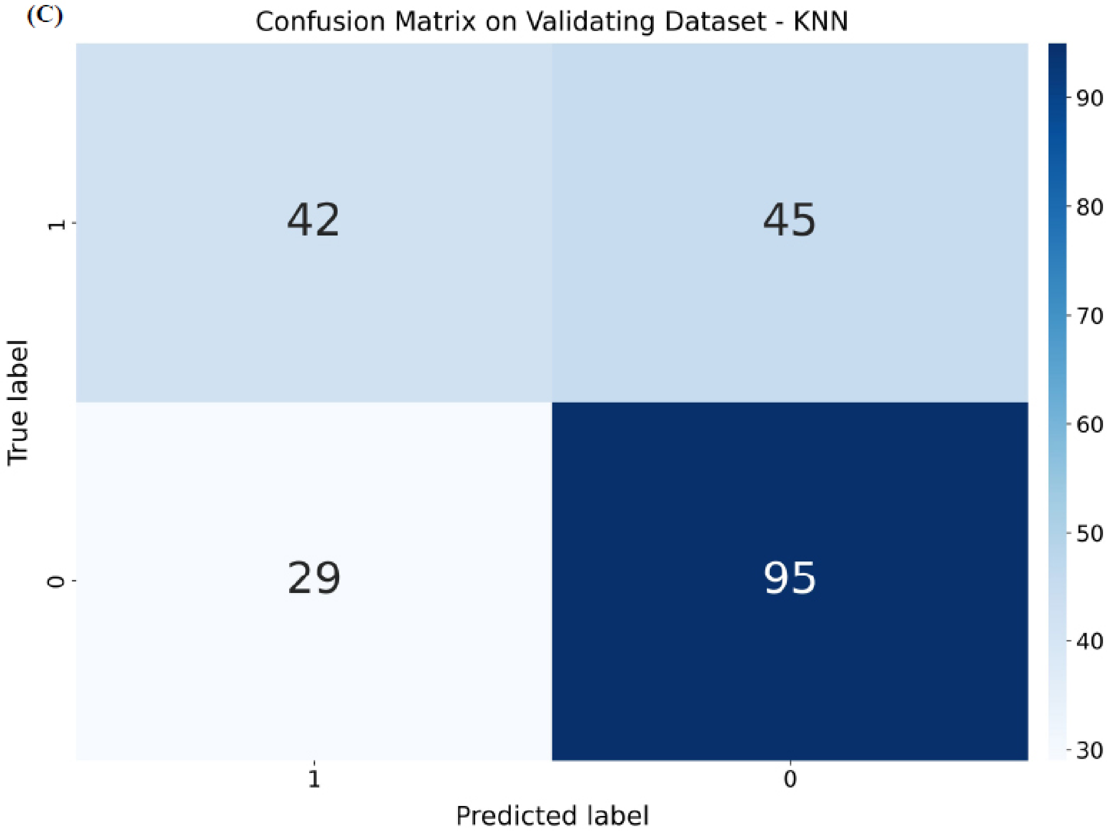

Fig. S3 contd.....

(D)

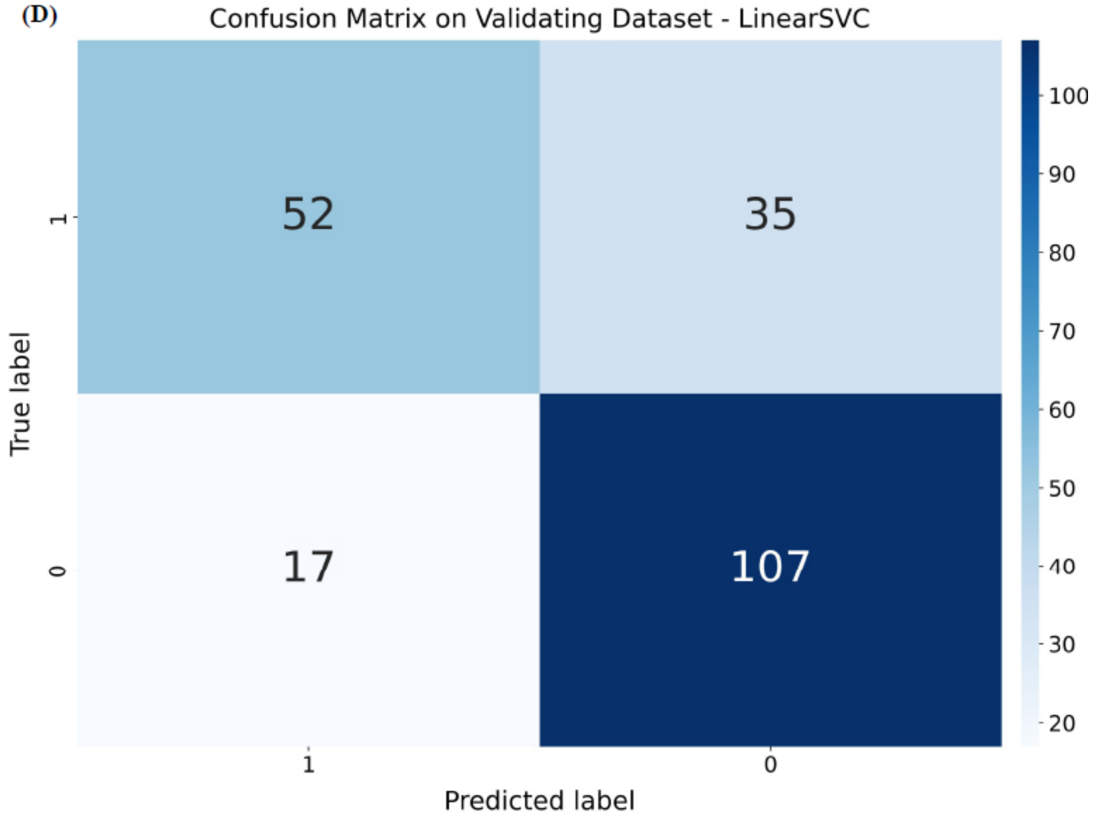

Fig. (S3). The Confusion Matrix of logistic regression (**A**), decision tree (**B**), KNN (**C**) and LinearSVC (**D**) in the validation set, respectively.
Notes: KNN, k-nearest neighbors; LinearSVC, linear support vector classification.

© 2025 The Author(s). Published by Bentham Science Publisher.

This is an open access article distributed under the terms of the Creative Commons Attribution 4.0 International Public License (CC-BY 4.0), a copy of which is available at: https://creativecommons.org/licenses/by/4.0/legalcode. This license permits unrestricted use, distribution, and reproduction in any medium, provided the original author and source are credited.
